# Age-related changes in relative expression stability of commonly used housekeeping genes in selected porcine tissues

**DOI:** 10.1186/1756-0500-4-441

**Published:** 2011-10-24

**Authors:** Muhammad Jasim Uddin, Mehmet Ulas Cinar, Dawit Tesfaye, Christian Looft, Ernst Tholen, Karl Schellander

**Affiliations:** 1 Animal Breeding and Husbandry/Genetics group, Institute of Animal Science, University of Bonn, Endenicher Allee 15, 53115 Bonn, Germany; 2 Department of Medicine, Faculty of Veterinary Science, Bangladesh Agricultural University, Mymensingh-2202, Bangladesh

## Abstract

**Background:**

Gene expression analysis using real-time RT-PCR (qRT-PCR) is increasingly important in biological research due to the high-throughput and accuracy of qRT-PCR. For accurate and reliable gene expression analysis, normalization of gene expression data against housekeeping genes or internal control genes is required. The stability of reference genes has a tremendous effect on the results of relative quantification of gene expression by qRT-PCR. The expression stability of reference genes could vary according to tissues, age of individuals and experimental conditions. In the pig however, very little information is available on the expression stability of reference genes. The aim of this research was therefore to develop a new set of reference genes which can be used for normalization of mRNA expression data of genes expressed in varieties of porcine tissues at different ages.

**Results:**

The mRNA expression stability of nine commonly used reference genes (*B2M, BLM, GAPDH, HPRT1, PPIA, RPL4, SDHA, TBP *and *YWHAZ*) was determined in varieties of tissues collected from newborn, young and adult pigs. geNorm, NormFinder and BestKeeper software were used to rank the genes according to their stability. geNorm software revealed that *RPL4, PPIA *and *YWHAZ *showed high stability in newborn and adult pigs, while *B2M, YWHAZ *and *SDHA *showed high stability in young pigs. In all cases, *GAPDH *showed the least stability in geNorm. NormFinder revealed that *TBP *was the most stable gene in newborn and young pigs, while *PPIA *was most stable in adult pigs. Moreover, geNorm software suggested that the geometric mean of three most stable gene would be the suitable combination for accurate normalization of gene expression study.

**Conclusions:**

Although, there was discrepancy in the ranking order of reference genes obtained by different analysing software methods, the geometric mean of the *RPL4, PPIA *and *YWHAZ *seems to be the most appropriate combination of housekeeping genes for accurate normalization of gene expression data in different porcine tissues at different ages.

## Background

The pig is one of the most studied organism in research community as a food as well as a model animal, and many projects in pigs require the quantification of genes for many purposes. Real-time quantitative PCR (qRT-PCR) is the most frequently used method for gene quantification nowadays. qRT-PCR is an efficient method for quantification of mRNA transcript levels due to its high sensitivity, reproducibility and large dynamic range. Furthermore, it is fast, easy to use and provides simultaneous measurement of gene expression in many different samples for a limited number of genes [1-3]. In case of qRT-PCR, when analyzing data for relative quantification, results are normalized to a reference. The most accepted approach to mRNA quantification is normalization of the expression level of a gene of interest (target gene) to the expression level of an internal stably expressed gene (control gene) [4-6]. The control gene, often termed reference gene or housekeeping gene, is a stably expressed gene that is experimentally verified in given species and tissues under given experimental conditions [3,7-9]. Normalizing to a reference gene is a widely used method because it is simple in theory. The normalization adjusts for differences in the quality or quantity of template RNA or starting material and differences in RNA preparation and cDNA synthesis, since the reference gene is exposed to the same preparation steps as the gene of interest. This allows the direct comparison of normalized transcript expression levels between samples. However, this approach requires the selection of at least one reference gene for validation of a corresponding qRT-PCR method. Normalization is extremely important to allow accurate comparison of the results between different samples and conditions in gene expression studies [4]. For instance, the commonly used reference genes such as *GAPDH *and β-actin are unfortunately often used without prior validation of their expression stability under the specific study conditions, but a number of studies have shown that the expression of those genes is significantly altered in some experimental conditions [10-12]. It is therefore necessary to validate the expression stability of reference genes prior to their use in an experimental protocol. Recently it has been recommended that a combination of reference genes should be used to obtain a more stable reference [6] and the use of a single reference gene is nowadays discouraged by more and more authors [4,6,13]. Because, a variability or alteration in the chosen reference gene by the experiment, however, may change the obtained results entirely and could be incorrect. Therefore, the validation of potential reference genes is essential.

An ideal reference gene should be stably expressed and unaffected by experimental protocol or status [14]. But, the recent studies showed that the housekeeping gene expressions could be changed according to the type of tissues [3,8,15] breeds [15], experimental condition (such as treatment or disease) [16-19] and age [15,20,21]. A set of reference genes are suggested on the basis of their stability over tissues in pigs [3,7,15,22,23] but studies for expression stability of housekeeping genes in varieties of porcine tissue collected from different age of pigs are rare. Therefore, this study was aimed to explore the expressions of nine mostly used housekeeping genes in 14 different tissues collected from three different ages of pigs (1 day old piglet, 2 months old young and 5 months old adult pigs) in order to select the suitable set of housekeeping genes that could be used as an internal control to normalize gene expression in pigs.

## Methods

### Tissues collection

A total of nine clinically healthy pigs of three age group were selected: neonatal (one day old), young (2 months old) and adult (5 months old) for this experiment. Each age group consisted of three animals of Pietrain, and all the animals were male and from the same batch. All pigs were kept at the Frankenforst experimental research farm at the University of Bonn (Germany). The animals were reared and slaughtered according to the rules of German performance stations [24]. The animals were fed same diet *ad libitum *during the whole experimental period. Blood was collected for peripheral blood mononuclear cells (PBMC) isolation. Lymph nodes (cervical and mesenteric), intestinal mucosa from duodenum, jejunum and ileum, tissues from stomach, liver, spleen, thymus, lung, kidney, heart and skin from ear were collected for mRNA isolation after slaughter. For mRNA isolation from tissues, samples were directly put into liquid nitrogen after washing in PBS. PBMC was isolated from whole blood using Ficoll-Histopaque (Sigma) following manufacturer's protocol. All samples were kept in -80°C till used.

### RNA isolation and cDNA synthesis

Total RNA was isolated from individual samples by using Tri-Reagent (Sigma-Aldrich, Munich, Germany) according to the standard protocol. In brief, sample was first grinded in a mortar, then mixed and homogenized with 1 ml Tri-Reagent using electric homogenizer. To ensure complete dissociation of nucleoprotein complexes, the sample was allowed to stand for 5 min before adding 0.2 ml of chloroform. The mixture was shaken and left at room temperature for 10 min and centrifuged at 12,000 × g for 15 min at 4°C. The upper aqueous phase was transferred to another fresh centrifuge tube and RNA was precipitated with 0.5 ml of isopropanol. After being incubated at room temperature for 10 min, the sample was centrifuged at 12,000 × g for 10 min at 4°C to get the RNA pellet, which was subsequently washed by 75% (v/v) ethanol. Centrifugation was then performed and the RNA pellet was air-dried and resuspended in 25 μl of DEPC treated water. RNA was isolated from PBMC using Picopure RNA isolation kit (Cat.# KIT0202; Arcturus). All samples were kept at -80°C until cleanup.

In order to remove possible contaminating genomic DNA, the extracted RNA was treated with 5 μl RQ1 DNase buffer, 5 units DNase and 40 units of RNase inhibitor in a 40 μl reaction volume. The mixture was incubated at 37°C for 1h followed by purification with the RNeasy Mini Kit (Qiagen, Hilden, Germany). Concentration of clean-up RNA was determined spectrophotometrically by using the NanoDrop (ND-8000) instrument; the purity of RNA was estimated by the ratio A260/A280 with respect to contaminants that absorb in the UV. Additional examination of integrity was done by denaturing agarose gel electrophoresis and ethidium bromide staining. Finally, the purified RNA was stored at -80°C for further analysis.

Approximately 1.5 μg of total RNA for each sample was transcribed into cDNA. cDNA was synthesised using GoScript (Cat.#A5000) reverse Transcription System (Promega, Germany) combined with OligoDT_15 _Primers, Recombinant RNasin^® ^Ribonuclease Inhibitor and GoScript™ Reverse Transcriptase according to the manufacturer's specification and protocol. cDNA was stored at -80°C until further use.

### Selection of reference genes and primer design

Only few previous studies validated selected reference genes across selected tissues in pigs [3,7,15,22,23] with specific purpose but no study was devoted to validate reference genes in the different tissues collected from different ages of pigs. However, 'traditional' reference genes like *GAPDH *and *TBP *have been most often used in pigs [3,22,23,25-29]. Regarding porcine organs, *ACTB, B2M, GAPDH, HMBS, HPRT1, RPL4, SDHA, TBP *and *YWHAZ *have been previously compared [3]. More specifically in recent days, *GAPDH, ACTB, RPL27, RPS29, RPS13 *are compared in porcine stomach [29]; *GAPDH, TBP, HPRT, RPS29, ACTB *and *RPL27 *are validated in porcine adipose tissues in different breeds of pigs [23] and *B2M, SDHA, ACTB, GAPDH, HPRT1 *and *TBP *expression stability are compared in porcine muscle and liver tissues in pigs [15]. The genes used in our study were selected based on these previous studies. Information about the nine candidate reference genes used in the present study is shown in table 1. The following nine commonly used reference genes were selected: *ACTB, GAPDH, HPRT1, B2M, SDHA, RPL4, YWHAZ, TBP *and *PPIA*. Primers were designed using the publicly available web-based Primer3 program [30] and are listed in table 1. They were tested using a BLAST analysis against the NCBI database (http://www.ncbi.nlm.nih.gov/tools/primer-blast).

**Table 1:** Selected candidate reference genes, primers, and PCR reactions efficiencies

Gene name	GeneBank accession number	Primer sequence (forward/reverse)	Amplicon length (bp)	Amplification efficiency (%)	**R**^**2**^	Average Ct of cDNA		
						**1 Day**	**2 months**	**5 months**

B2M	NM_213978.1	ACTTTTCACACCGCTCCAGT CGGATGGAACCCAGATACAT	180	86.83	0.999	20.23	19.24	20.63

BLM	NM_001123084.1	TCCTCACCTTCTGCATTTCC GTGGTGGCTGAGAATCCTGT	152	95.94	0.995	25.29	24.12	24.89

GAPDH	AF017079.1	ACCCAGAAGACTGTGGATGG ACGCCTGCTTCACCACCTTC	247	95.95	0.991	26.82	26.22	26.29

HPRT1	NM_001032376.2	AACCTTGCTTTCCTTGGTCA TCAAGGGCATAGCCTACCAC	150	81.88	0.997	22.27	21.28	22.29

PPIA	NM_214353.1	CACAAACGGTTCCCAGTTTT TGTCCACAGTCAGCAATGGT	171	82.96	0.995	16.82	16.31	17.61

RPL4	DQ845176.1	AGGAGGCTGTTCTGCTTCTG TCCAGGGATGTTTCTGAAGG	185	91.07	0.995	16.65	16.80	17.32

SDHA	DQ178128.1	AGAGCCTCAAGTTCGGGAAG CAGGAGATCCAAGGCAAAAT	149	86.41	0.989	20.55	20.64	22.34

TBP	DQ178129.1	ACGTTCGGTTTAGGTTGCAG GCAGCACAGTACGAGCAACT	118	99.59	0.995	24.44	23.92	24.31

YWHAZ	DQ178130.1	ATTGGGTCTGGCCCTTAACT GCGTGCTGTCTTTGTATGACTC	146	93.83	0.997	20.35	19.64	19.92

*R^2^, correlation coefficient of the slope of the standard curve

### qReal-Time PCR

Nine-fold serial dilution of plasmids DNA were prepared and used as template for the generation of the standard curve. In each run, the 96-well microtiter plate contained each cDNA sample, plasmid standards for the standard curves and no-template control. A no-template control (NTC) was included in each run for each gene to check for contamination. Quantitative real-time RT-PCR (qRT-PCR) was set up using 2 μl first-strand cDNA template, 7.4 μl deionized H_2_O, 0.3 μM of upstream and downstream primers and 10 μl 1× Power SYBR Green I master mix with ROX as reference dye (Bio-Rad). The thermal cycling conditions were 3 min at 95°C followed by 15 s at 95°C (40 cycles) and 1 min at 60°C. Experiments were performed using the StepOnePlus™ Real-Time PCR System (Applied Biosystems). Based on the Ct values for all dilution points in a series, a standard curve was generated using linear regression and the slope and the PCR amplification efficiency of each primer pair is calculated from the slope of a standard curve [8]. Melting curve analysis was constructed to verify the presence of gene-specific peak and the absence of primer dimer. Agarose gel electrophoresis was performed to test for the specificity of the amplicons. To ensure repeatability of the experiments, all the reactions were executed in triplicate and the average was used for further analysis.

### Determination of reference gene expression stability

The raw qRT-PCR amplification data was exported from the StepOne^® ^software (Applied Biosystem) to Microsoft^® ^Excel. The averages of the Ct-values for each triplicate were used for stability comparison of candidate reference genes in the NormFinder, geNorm and BestKeeper. The Proc GLM (ver9.2; SAS, SAS Institute Inc., Cary, NC, USA) analysis was performed to detect the effect of age and organs on the expression of housekeeping genes. Differences in gene expression levels between age groups within tissues were determined using t-test in SAS. *P *< 0.05 was considered statistically significant.

Ct values of all samples were exported to Excel, ordered for use in geNormPlus software (15 days free trial version qBasePlus; http://www.biogazelle.com) and transformed to relative quantities using the gene-specific PCR amplification efficiency [31]. These relative quantities were then exported to geNormPlus to analyze gene expression stability [6]. The approach of reference gene selection implemented in geNorm relies on the principle that the expression ratio of two ideal reference genes should be identical in all samples, independent of the treatment, condition, or tissue type. Increasing variations in the expression ratio between two genes correspond to lower expression stability across samples. geNorm calculates the stability using a pairwise comparison model [6] and determines the level of pairwise variation for each reference gene with all other reference genes as the standard deviation of the logarithmically transformed expression ratios. In this way, the reference gene expression stability measure (*M *value) was calculated as the average pairwise variation of a particular gene with all other control genes included in the analysis [6,8]. Lower *M *values represent higher expression stabilities, whereas the least stable gene showed the highest *M *value generates a ranking of genes according to their *M *values resulting in the identification of the genes with the most stable expression in the samples under analysis. geNorm was also used to estimate the normalization factor (NF_*n*_) by calculating the geometric mean of the expression levels of the *n *best reference genes [6]. The optimisation of the number of reference genes starts with the inclusion of the two genes with the lowest *M *value, and continues by sequentially adding genes with increasing values of *M*. Thus, geNorm calculates the pairwise variation V_*n*_/V_*n*+1 _between two sequential normalization factors NF_*n *_and NF_*n*+1 _containing an increasing number of reference genes [6]. A large variation means that the added gene has a significant effect on the normalization and should preferably be included for calculation of a reliable normalization factor. Ideally, extra reference genes are included until the variation V_*n*_/V_*n*+1 _drops below a given threshold. According to the geNorm, if V_n_/V_n+1 _< 0.15 the inclusion of an additional reference gene is not required and the recommended number of reference genes is given by *n *[6].

NormFinder uses an ANOVA-based model [32]. The software calculates a stability value for all candidate reference genes tested. The stability value is based on the combined estimate of intra- and inter-group expression variations of the genes studied [32]. For each gene, the average Ct value of each triplicate reaction was converted to relative quantity data as described for geNorm, to calculate the stability value with NormFinder program [32]. The NormFinder reference tool was applied to rank the candidate reference gene expression stability for all samples with no subgroup determination as well as with age as subgroup. A low stability value, indicating a low combined intra- and inter-group variation, indicates high expression stability [32].

The average Ct value of each triplicate reaction was used (without conversion to relative quantity) to analyze the stability value of studied genes via BestKeeper [33] which creates a pairwise correlation coefficient between each gene and the BestKeeper index (BI). This index is the geometric mean of the Ct values of all candidate reference genes. BestKeeper also calculates standard deviation (SD) of the Ct values between the whole data set. The gene with the highest coefficient of correlation with the BI indicates the highest stability [33].

## Results

### Purity, quantity of extracted RNA and verification of amplicons

**Figure 1:**
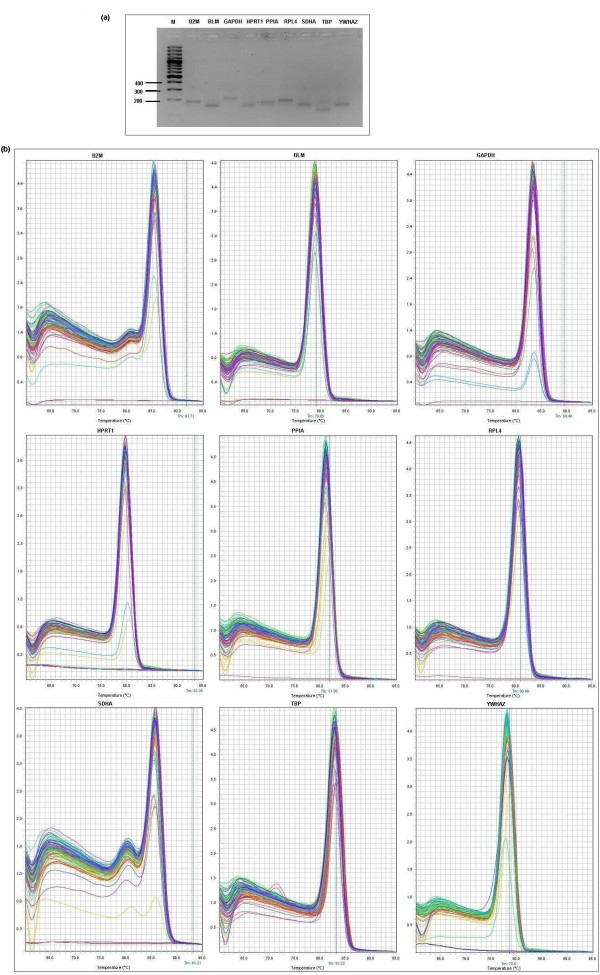
**Confirmation of amplicon size and primer specificity of studied genes **. (a) Agarose gel electrophoresis showing specific reverse transcription PCR products of the expected size for each gene, M represents DNA size marker. (b) Melting curves generated for all genes.

The optical density (OD) ratio A260/A280 nm measured with a Nanodrop spectrophotometer was 1.95 ± 0.16 (OD A260/A280 ratio ± SD). The average RNA concentration after extraction using the Tri-reagent (for tissues) and PicoPure (for PBMC) was 1.65 μg/μl ± 1.03 (μg/μl ± SD). The results of the averaged amplification efficiencies are shown in table 1. The amplification efficiencies for the nine candidate reference genes ranged between 81.88% and 99.59%. The agarose gel electrophoresis (figure 1a) and melting curve analysis (figure 1b and table 1) revealed that all primer pairs amplified a single PCR product with expected size. Furthermore, sequence analysis of cloned amplicons revealed that all sequenced amplified fragments were identical to sequences used for primer design from GenBank.

### Expression levels of candidate reference genes

**Figure 2:**
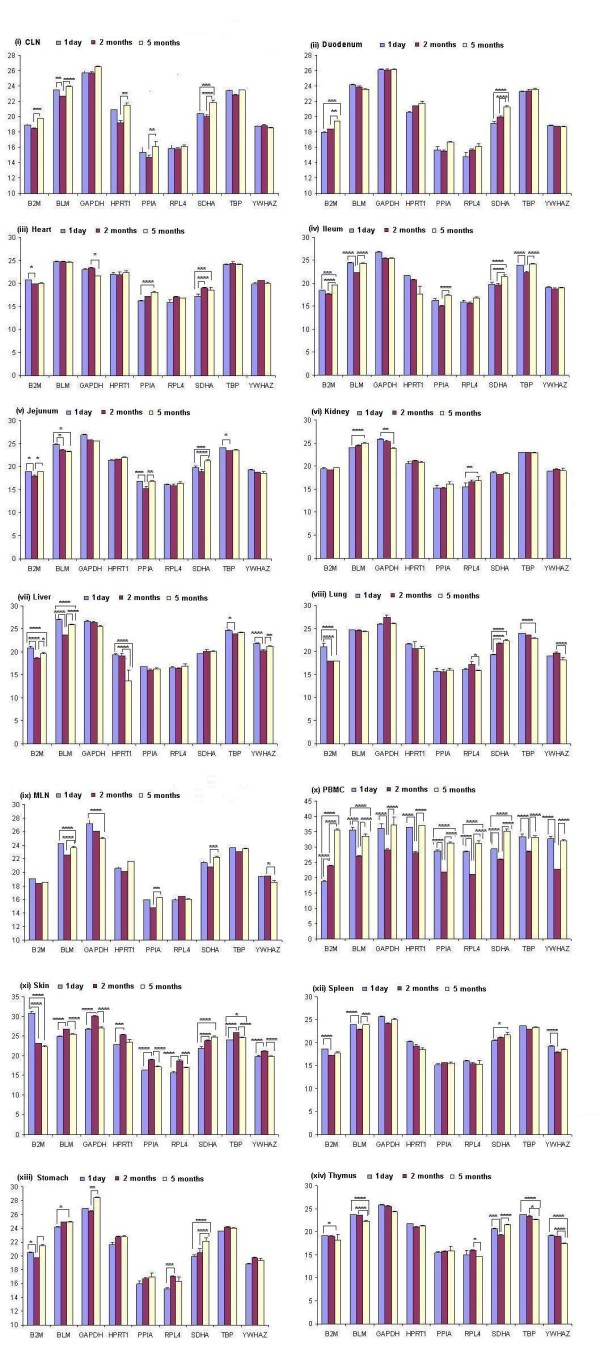
**Average cycle threshold (Ct) values of candidate reference genes tested in porcine tissues at different ages **. The values are the average qRT-PCR cycle threshold numbers (Ct values). The bars indicate standard deviation. *P < 0.05; **P < 0.01; ***P < 0.001 and ****P < 0.0001

The cycle threshold (Ct) values obtained throughout the study were low enough to pursue the analysis reliably: Overall (by combining Ct values of all ages for each gene), out of the nine genes studied, *PPIA *(mean Ct 16.91) and *RPL4 *(mean Ct 16.92) were expressed at the highest levels, followed by *YWHAZ *(mean Ct 19.97), *B2M *(mean Ct 20.03), *SDHA *(mean Ct 21.17) and *HPRT1 *(mean Ct 22.05). *GAPDH *(mean Ct 26.44) was expressed at the lowest level in the porcine tissues used in this study (Additional file 1: Table S1). When expression values were compared between ages within a tissue, the mRNA expression (average Ct values) differences for *B2M *and *SDHA *were significant (*P *< 0.05) between ages in 12 tissues, *BLM *mRNA difference was significant (*P *< 0.05) between ages in 11 tissues and the mRNA differences for *GAPDH, PPIA, TBP *and *YWHAZ *were significant (*P *< 0.05) between ages in seven tissues out of 14 tissues (figure 2). In case of PBMC and skin, all the candidate reference genes were expressed differentially (*P *< 0.05) between ages (figure 2x and 2xi). According to the Ct values for candidate genes, less expression variability could be seen in duodenum (figure 2ii) followed by kidney (figure 2vi), spleen (figure 2xii) and heart (figure 2iii). Moreover, the expression of reference genes was found to be influenced by organ, age and age-organ interaction (Additional file 2: Table S2).

### Identification of optimal reference genes

**Figure 3:**
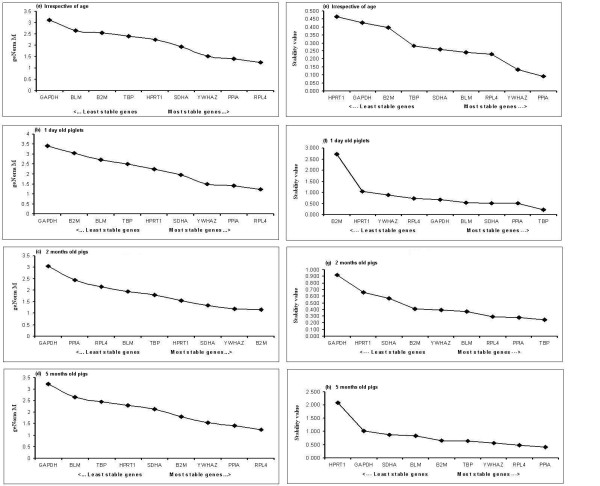
**Ranking of nine candidate reference genes using geNorm and NormFinder softwares **. (a-d) geNorm ranks the candidate reference genes based on their stability parameter *M*. The lower the *M *value, the higher the expression stability. (e-h) NormFinder ranks the genes based on a calculated stability value. The lower the stability value, the higher the expression stability.

Figure 3a and 3e show the ranking of the nine candidate reference genes across the tissues without considering ages of individuals based on their stability values calculated using geNorm and NormFinder, respectively. Both softwares showed that *RPL4, PPIA *and *YWHAZ *are the most stable genes. Similar stability for candidate genes could also be found in tissues collected from 5 months adult pigs (figure 3d and 3h). However, the expression stability was never consistent between the used softwares. geNorm showed that *RPL4 *was the most stable candidate reference gene followed by *PPIA *and *YWHAZ *in tissues collected from 1 day old piglets (figure 3b), whereas *B2M *was the most stable reference gene followed by *YWHAZ *and *SDHA *in case of 2 months old young pigs (figure 3c). *GAPDH *has the highest stability value in all ages group when expression stability was analyzed using geNorm (figure 3a-d). On the other hand, NormFinder showed that *PPIA *is the most stable gene when all tissues were considered together and in tissues collected from 5 months old adult pigs (figure 3e, h), whereas *TBP *showed highest stability in tissues collected from 1 day old piglet and in 2 months old young pigs (figure 3f, g). Additionally, *BLM *and *RPL4 *were recommended as the best combination of two genes with the stability value 0.083, while *PPIA *was recommended as the best gene with stability value 0.091 by NormFinder. Figure 3a-d shows the ranking of the nine candidate reference genes based on their *M *value calculated using geNorm. In all age groups, the most stable three candidate reference genes started with an *M *value below or equal to 1.5, which is the default limit below which candidate reference genes can be classified as stably expressed.

The results of reference gene evaluation by the BestKeeper tool are shown in table 2. According to the variability observed, candidate reference genes can be identified as the most stable genes exhibiting the lowest coefficient of variance (CV ± SD). In this context, we found that *YWHAZ *is the most stable reference gene in tissues collected from 2 months old young pigs (table 2). It is important to note that, genes that show a SD higher than 1 should be considered unacceptable [33,34]. A low SD of the cycle threshold (Ct) values should be expected for a useful reference gene. In this study, the estimation of the SD (± Ct) of the CV [%Ct] values for all the genes except *YWHAZ *at 2 months (bold italic letters; table 2), was higher. This constitutes a reason to exclude these genes from the BestKeeper index calculation, as they are not reliable reference candidate gene in this setting [33].

**Table 2:** Expression stability of nine candidate reference gens evaluated by BestKeeper software

	B2M	BLM	GAPDH	HPRT1	PPIA	RPL4	SDHA	TBP	YWHAZ	BK
*Irrespective of age*										

n*	42	42	42	42	42	42	42	42	42	42

SD [± Ct]	1.91	1.36	1.56	2.12	1.69	1.55	1.90	1.19	1.56	1.49

CV [% Ct]	9.54	5.50	5.90	9.67	9.99	9.16	8.95	4.92	7.81	7.07

*1day*										

n**	42	42	42	42	42	42	42	42	42	42

SD [± Ct]	1.86	1.70	1.42	2.11	1.70	1.69	1.61	1.30	1.99	1.47

CV [% Ct]	9.17	6.70	5.28	9.45	10.11	10.17	7.82	5.30	9.76	6.95

*2momths*										

n**	42	42	42	42	42	42	42	42	42	42

SD [± Ct]	1.37	1.10	1.19	1.73	1.35	1.05	1.49	1.04	***0.96***	1.11

CV [% Ct]	7.13	4.55	4.54	8.02	8.30	6.24	7.23	4.36	4.89	5.38

*5 months*										

n**	42	42	42	42	42	42	42	42	42	42

SD [± Ct]	2.49	1.47	2.01	2.86	2.04	2.00	2.21	1.31	1.92	1.92

CV [% Ct]	12.07	5.92	7.65	13.12	11.56	11.57	9.89	5.39	9.64	8.97

Descriptive statistics of nine candidate reference genes based on their cycle threshold (Ct) values. In the last column the BestKeeper (BK) index is computed together with descriptive parameters for the nine genes. Abbreviations: CV [%Ct]: the coefficient of variance expressed as a percentage on the Ct level; SD [± Ct]: the standard deviation of the Ct; Results from overall tissues irrespective of age and in different ages (1 day, 2 months and 5 months) are shown. * indicates the number of samples (since BestKeeper tool has limitation for 100 samples, the average Ct for three individuals was used for analysis); ** indicates the average for triplicate run was used for analysis.

### Determination of the optimal number of reference genes for normalization

**Figure 4:**
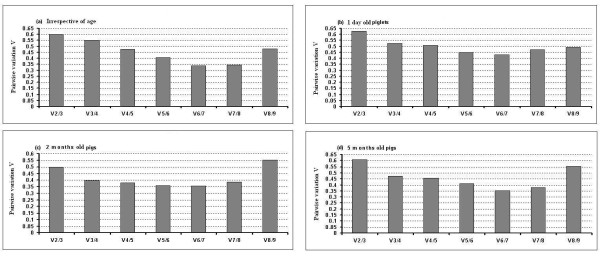
**Determination of the optimal number of reference genes for normalization **. The geNorm software calculates the normalization factor from an increasing number of genes (starting with at least two) for which the variable V defines the pairwise variation between two sequential normalization factors. The lower the pairwise variation, the better is the combination of genes for reference. V6/7 for example, shows the variation between the normalization factors of six genes in relation to seven genes and shows that six genes is the combination providing the lowest pairwise variation.

**Figure 5:**
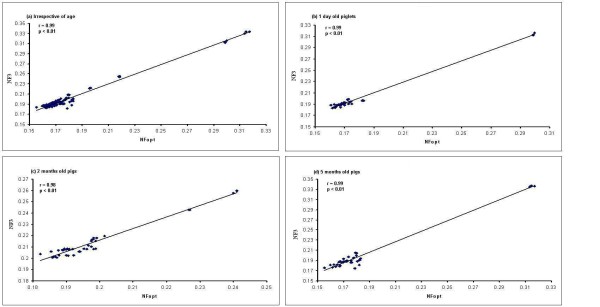
**Correlation between the NF of most three stable and optimal number endogenous control **. Pearson's correlations between the NFs of three endogenous control genes (NF3) and optimal number (six) of endogenous control genes (NFopt) for (a) all samples irrespective of age, (b) all tissues collected from 1 day old piglets, (c) all tissues collected from 2 months old young pigs, and (d) all tissues collected from 5 months old adult pigs.

In addition to the stability results, the geNorm software can determine the optimal number of reference genes necessary to calculate a normalization factor (NF). The results are shown in figure 4. In all the cases in this study, V6/7 (the variation between the normalization factors of six genes in relation to seven genes) showed the lowest pairwise variation indicated that six genes is the optimal number of reference genes for normalization. As shown in figure 4a to 4d, 6 endogenous control genes are necessary to obtain the lowest changing V values in all analyzed samples. However, it is impractical to use excessive numbers of endogenous control genes for normalization, particularly when only a small number of target genes need to be studied or for rare samples that are very difficult to acquire [6,22]. Therefore, the use of the three most stable housekeeping genes for the calculation of the NF was considered acceptable for the majority of experiments [6,22]. To verify that the use of three housekeeping genes simultaneously is adequate for normalization of qRT-PCR, the correlation of NF values between the geometric means of the three most stable genes and the optimal number of genes was calculated for all sample groups. As shown in figure 5, there is a very good correlation between the two NF measures (i.e., the theoretical optimal number and proposed number, three) for all 14 samples in all ages including overall tissues irrespective of age (*r *= 0.99 to 0.98, Pearson) (figure 5a to 5d). This result demonstrates that the three most stable housekeeping genes are sufficient for an accurate normalization of our qRT-PCR data [6,22]. In addition, there is a very good agreement between geNorm and NormFinder softwares identifying three out of six most stable genes, namely *RPL4, PPIA *and *YWHAZ*. We therefore in general postulate that the combination of *RPL4, PPIA *and *YWHAZ *is the most appropriate normalization approach for gene expression studies in different tissues from pigs at different ages.

## Discussion

For an exact comparison of mRNA transcription in different samples or tissues it is crucial to choose the appropriate reference gene. The optimal reference gene should be constantly transcribed in all types of cells at any time in cell cycle and differentiation. Moreover the transcription of such a gene should not be regulated by internal or external influences, at least not more than the general variation in RNA synthesis [3]. The reference gene used for normalization of gene expression in qRT-PCR studies should also pass through the same steps of analysis as the gene to be quantified. However, such a perfect reference gene does probably not exist. Recent research has demonstrated that the expression of housekeeping genes may be altered due to differences in tissue types [3,15,22], breeds [23], ages [21,23] and experimental condition or treatment [6,16-19]. Therefore, it is critical to elucidate differences that may exist in housekeeping genes between younger and older adults. As an increasing volume of data continues to be published exploring mRNA expression in cases of age-depended disease, there has been a greater interest in evaluating the commonly used, widely expressed housekeeping genes for comparisons between ages. Without this information, age-dependent comparisons are very difficult to make. Therefore, it is necessary to investigate the validity and reliability of measuring the expression of various housekeeping genes in porcine tissues at different ages using qRT-PCR. To the author's knowledge, this study is the first to report that aging can influence the expression of certain housekeeping genes in pigs.

Numerous studies have been carried out in order to evaluate reference genes in specific tissues in several species. The majority of these studies are directed towards specific tissues in pigs [3,7,29,35,36]. Taken together, it is very difficult to find a 'universal' reference gene having stable expression in all cell types and tissues, and in particular to find reference genes that remain stable between samples taken at different ages under different experimental conditions. According to the NCBI-PubMed statistics [22], *GAPDH *and *ACTB *are the two mostly used porcine housekeeping genes. But they have been shown to vary considerably and are consequently unsuitable as reference genes for normalization of gene expression analysis in some cases [10-12]. Also the low expressed reference gene *TBP *is highly regulated in pigs [36]. The first priority, however, is to identify genes with stable expression preferably across cell types since many qRT-PCR studies are performed on cDNA isolated from tissues with a mixed cell population. Presently, only few major publications describe the stability of housekeeping genes in pig and are based on limited samples of specific categories [3,7,29,35,36]. Our comprehensive set of representative tissue samples and selected housekeeping genes provide valuable recommendations for the choice of endogenous control genes for the study of gene expression patterns in normal tissues. Notably, our results coincided with the finding of Gu et al [22] reported that *YWHAZ *is one of the most stably expressed reference genes across tissues in healthy pigs. Nygard et al. [3] reported that *RPL4, TBP *and *YWHAZ *have the highest stability across tissues collected from healthy pigs which are in good agreement with our findings. In this study, geNorm showed that *PPIA, YWHAZ *and *RPL4 *are the most stable housekeeping genes across tissues in case of newborn piglets, adults and in irrespective of ages. Additionally, *PPIA, RPL4 *and *YWHAZ *are detected to be the most stably expressed genes across the tissues by NormFinder.

geNorm finding is contradictory to the findings of Erkens et al. [7] who reported that *TBP *is one of the most stable housekeeping gene in porcine backfat and muscle (longissimus dorsi) while *SDHA *is reported as an unstable gene. Kuijk et al. [36] reported that *GAPDH *and *B2M *are the most and least stably genes, respectively in porcine oocytes and perimplantation embryos. Although tissues are different, the finding of this study is in good agreement with Piorkowska et al [23] who recently reported that *GAPDH *is the least stable reference candidate gene in porcine adipose tissues collected from different pig breeds. The findings of this study that commonly used housekeeping genes studied are expressed differentially across porcine tissues is supported by Svobodova et al [35] in pigs. Moreover, Svobodova et al [35] found that *GAPDH *expression was unstable across porcine tissues which is in good agreement with our result. Svobodova et al [35] reported that *HPRT1 *has the highest stability, whereas this study found that according to the geNorm *HPRT1 *is moderately stable across the porcine tissues (figure 3a-3d) but unstable according to the NormFinder (figure 3e-3h). Pierzchala et al. [15] recently reported that *HPRT1 *and *TBP *are the most stable housekeeping genes in porcine liver and in three different muscle tissues which is partially supporting the NormFinder result as well as conflicting to the geNorm result. In this study we found that *HPRT1 *and *TBP *are moderately stable genes in geNorm analysis (figure 3a-3d), whereas *TBP *is a stable gene but *HPRT1 *is an unstable gene in NormFinder analysis (figure 3e-3h). *RPL4, HPRT1 *and *B2M *are reported as stably expressed and suitable candidate genes in intestinal tissues collected from healthy pig and from pigs with enteritis [37]. Reportedly, *GAPDH *is the least stable gene while *RPL27 *is most stable housekeeping gene in porcine stomach tissue [29]. However, different housekeeping genes are identified between the previous studies and our study, as the samples varied in their cell, tissue, sex and developmental stage specificities, and different catalogues of selected housekeeping genes are chosen.

According to the BestKeeper analysis software, all the studied reference candidate genes, except *YWHAZ *at 2 months old young pigs tissues, are less suitable. Several studies previously reported similar findings for BestKeeper [8,29,34] and few studies followed the BestKeeper analysis method compared to geNorm and NormFinder. It is important to note that very similar discrepancies between the different algorithms have been observed in previous studies comparing statistical analysis methods [8,16,29,34,38,39]. However, we found that the first three most stable reference genes in most cases were consistently the same when using geNorm and NormFinder, even if they were not in the exact same ranking order. Similar findings are reported by previous studies in horse, human and plants [8,16,38,40]. Such discrepancy could be explained by genes' coregulation. Indeed, coregulated genes may become highly ranked independently of their expression stabilities with geNorm software [32]. Moreover, NormFinder takes into account variation across subgroups, thus avoiding artificial selection of coregulated genes by analyzing the expression stability of candidate genes independently from each other [6]. However, no studies dealing with porcine reference genes stability used different analysis methods except geNorm [3,7,22,23,29].

As described above, geNorm also provides a measure for the best number of reference genes that should be used for optimal normalization. In agreement with several previous studies, we postulate that the use of more than one reference gene allows for a more accurate normalization than the use of only one reference gene [4,6,16,22,32]. Based on a cut-off point for the V value, as described by Vandesompele et al [6], a combination of the six most stable reference genes was calculated as being optimal for gene expression studies in different porcine tissues over ages (figure 4). However, as we described above and other studies [6,22] recommended, the combination of the most three stable genes seems to be appropriate for accurate normalization.

## Conclusion

This investigation found evidence that there can be variafition in the expression of commonly used housekeeping genes with populations of different ages. Due to the new influx of data suggesting alterations in mRNA expression according to ages, we feel that beside therapy uses or experimental condition, the selection of housekeeping genes based upon the age of populations used should be taken into consideration. This shows again that the choice of reference genes cannot be transposed from one study to the other without validation for the specifics of each experimental protocol. In general, we recommend using the geometric mean of *RPL4, PPIA *and *YWHAZ *to guarantee suitable normalization across the porcine tissues obtained from pigs of different ages.

## List of abbreviations

qRT-PCR: quantitative real-time reverse transcriptase polymerase chain reaction; *B2M*: beta-2-microglobulin; *BLM*: Bloom syndrome, RecQ helicase-like; *GAPDH*: glyceraldehyde 3-phosphate dehydrogenase; *HPRT1*: hypoxanthine phosphoribosyltransferase 1; *PPIA*: peptidylprolyl isomerase A (cyclophilin A); *RPL4*: ribosomal protein L4; *SDHA*: succinate dehydrogenase complex subunit A flavoprotein; *TBP*: TATA box binding protein; *YWHAZ*: tyrosine 3-monooxygenase/tryptophan 5-monooxygenase activation protein zeta polypeptide; NTC: no-template control; Ct: cycle threshold; SD: standard deviation; BI: BestKeeper Index.

## Competing interests

The authors declare that they have no competing financial or other interest in relation to this work.

## Authors' contributions

MJU performed the experiments, analyzed data and prepared and edited the manuscript. MUC, DT, ET and CL edited the manuscript with MJU. KS criticized the experimental design and edited the manuscript. All authors read and approved the final manuscript.

## Supplementary Material

Additional file 1 **Mean relative expression of candidate genes and effects of age and organ on expression level**. The average and SD of the Ct values for different candidate reference genes studied in different tissues collected from 1 day old piglets, 2 months old young and 5 months old adult pigs. Click here for file

Additional file 2 **Relative expression of candidate genes and effect of age and organ on expression level**. Overall expression data of reference candidate genes. Summary of the Proc GLM (ver.9.2; SAS, SAS Institute Inc., Cary, NC, USA) analysis detecting significant effects of age, organs and age-organ interaction on the expression of reference candidate genes. ***P<0.001. Click here for file
