# Supplementary material for: Age-related changes in relative expression stability of commonly used housekeeping genes in selected porcine tissues
Source: BMC Res Notes. 2011 Oct 24;4:441. doi: 10.1186/1756-0500-4-441 (PMC3219825; doi:10.1186/1756-0500-4-441)
Supplement: Additional file 1 — Mean relative expression of candidate genes and effects of age and organ on expression level. The average and SD of the Ct values for different candidate reference genes studied in different tissues collected from 1 day old piglets, 2 months old young and 5 months old adult pigs. [file 1756-0500-4-441-S1.DOC]

**Table S1. Mean relative expression of candidate genes and effects of age and organ on expression level.**

| Age | HKG | CLN (SD) | Duodenum (SD) | Heart (SD) | Ileum (SD) | Jejunum (SD) | Kidney (SD) | Liver (SD) | Lung (SD) | MLN (SD) | PBMC (SD) | Skin (SD) | Spleen (SD) | Stomach (SD) | Thymus (SD) |
| --- | --- | --- | --- | --- | --- | --- | --- | --- | --- | --- | --- | --- | --- | --- | --- |
| 1 day | B2M | 18.91(0.08) | 17.96 (0.04) | 20.75 (0.08) | 18.42 (0.11) | 18.85 (0.05) | 19.48 (0.15) | 20.92 (0.25) | 21.01 (0.80) | 19.05 (0.06) | 18.62 (0.32) | 30.92 (0.34) | 18.61 (0.09) | 20.54 (0.03) | 19.18 (0.09) |
|  | BLM | 23.48 (0.06) | 24.18 (0.09) | 24.81 (0.10) | 24.46 (0.06) | 24.94 (0.07) | 23.96 (0.02) | 26.99 (0.07) | 24.80 (0.02) | 24.24 (0.07) | 35.46 (0.96) | 24.92 (0.07) | 23.93 (0.04) | 24.18 (0.03) | 23.75 (0.07) |
|  | GAPDH | 25.74 (0.24) | 26.16 (0.03) | 22.99 (0.25) | 26.74 (0.12) | 26.90 (0.06) | 25.85 (0.17) | 26.68 (0.17) | 25.99 (0.17) | 27.13 (0.50) | 36.09 (1.50) | 26.77 (0.24) | 25.67 (0.14) | 26.86 (0.06) | 25.89 (0.06) |
|  | HPRT1 | 20.91 (0.08) | 20.56 (0.08) | 22.00 (0.30) | 21.68 (0.01) | 21.44 (0.14) | 20.58 (0.43) | 19.37 (0.22) | 21.70 (0.05) | 20.62 (0.27) | 36.46 (0.00) | 22.81 (0.13) | 20.24 (0.08) | 21.66 (0.29) | 21.75 (0.08) |
|  | PPIA | 15.40 (0.62) | 15.65 (0.51) | 16.26 (0.02) | 16.18 (0.49) | 16.80 (0.12) | 15.24 (0.63) | 16.77 (0.02) | 15.77 (0.62) | 15.93 (0.03) | 28.69 (0.36) | 16.35 (0.09) | 15.08 (0.32) | 15.94 (0.42) | 15.40 (0.23) |
|  | RPL4 | 15.87 (0.45) | 14.80 (0.53) | 15.86 (0.55) | 15.86 (0.46) | 16.16 (0.10) | 15.44 (0.86) | 16.59 (0.15) | 16.06 (0.29) | 15.97 (0.17) | 28.47 (0.21) | 15.73 (0.18) | 16.01 (0.07) | 15.28 (0.13) | 14.97 (1.02) |
|  | SDHA | 20.39 (0.12) | 19.15 (0.16) | 17.19 (0.52) | 19.82 (0.41) | 19.82 (0.33) | 18.51 (0.26) | 19.68 (0.02) | 19.29 (0.10) | 21.43 (0.24) | 29.39 (0.13) | 21.93 (0.31) | 20.49 (0.08) | 19.90 (0.27) | 20.68 (0.08) |
|  | TBP | 23.43 (0.13) | 23.27 (0.06) | 24.13 (0.04) | 23.85 (0.05) | 24.13 (0.01) | 22.96 (0.05) | 24.63 (0.12) | 23.96 (0.02) | 23.60 (0.05) | 33.32 (1.04) | 23.95 (0.04) | 23.61 (0.07) | 23.59 (0.04) | 23.75 (0.03) |
|  | YWHAZ | 18.82 (0.08) | 18.88 (0.04) | 19.93 (0.21) | 19.09 (0.10) | 19.36 (0.05) | 18.85 (0.07) | 21.87 (0.13) | 18.97 (0.01) | 19.36 (0.09) | 32.74 (0.69) | 19.87 (0.19) | 19.25 (0.06) | 18.84 (0.09) | 19.12 (0.17) |
| 2 months | B2M | 18.44 (0.08) | 18.38 (0.05) | 19.85 (0.06) | 17.70 (0.10) | 17.88 (0.33) | 19.20 (0.04) | 18.71 (0.04) | 17.92 (0.10) | 18.36 (0.13) | 23.80 (0.14) | 23.19 (0.02) | 17.17 (0.03) | 19.70 (0.05) | 19.00 (0.12) |
|  | BLM | 22.65 (0.03) | 23.89 (0.08) | 24.64 (0.27) | 22.30 (0.07) | 23.67 (0.08) | 24.50 (0.09) | 23.67 (0.05) | 24.58 (0.06) | 22.57 (0.03) | 27.05 (0.08) | 26.78 (0.06) | 22.94 (0.02) | 24.86 (0.03) | 23.60 (0.07) |
|  | GAPDH | 25.75 (0.11) | 26.03 (0.16) | 23.37 (0.05) | 25.50 (0.09) | 25.79 (0.15) | 25.31 (0.20) | 26.47 (0.22) | 27.42 (0.45) | 26.08 (0.22) | 29.05 (0.38) | 30.02 (0.23) | 24.24 (0.05) | 26.44 (0.16) | 25.57 (0.08) |
|  | HPRT1 | 19.19 (0.34) | 21.37 (0.05) | 21.91 (0.52) | 20.66 (0.21) | 21.61 (0.06) | 21.07 (0.17) | 19.09 (0.50) | 20.65 (1.45) | 20.13 (0.02) | 28.10 (0.37) | 25.31 (0.08) | 19.23 (0.30) | 22.77 (0.12) | 21.04 (0.09) |
|  | PPIA | 14.72 (0.25) | 15.52 (0.17) | 17.18 (0.04) | 15.03 (0.12) | 15.25 (0.56) | 15.21 (0.11) | 16.01 (0.21) | 15.58 (0.58) | 14.82 (0.24) | 21.77 (0.06) | 19.04 (0.11) | 15.65 (0.01) | 16.70 (0.18) | 15.78 (0.13) |
|  | RPL4 | 15.77 (0.13) | 15.69 (0.09) | 17.14 (0.06) | 15.69 (0.15) | 15.89 (0.33) | 16.50 (0.49) | 16.43 (0.14) | 17.27 (0.46) | 16.45 (0.61) | 21.09 (0.06) | 18.70 (0.22) | 15.48 (0.14) | 17.00 (0.15) | 16.05 (0.09) |
|  | SDHA | 19.97 (0.33) | 19.92 (0.23) | 18.95 (0.13) | 19.56 (0.39) | 18.93 (0.52) | 18.25 (0.03) | 20.11 (0.31) | 21.78 (0.09) | 20.84 (0.42) | 25.85 (0.08) | 23.81 (0.17) | 21.13 (0.14) | 20.47 (0.56) | 19.35 (0.13) |
|  | TBP | 22.87 (0.06) | 23.36 (0.22) | 24.39 (0.23) | 22.36 (0.20) | 23.41 (0.09) | 22.98 (0.05) | 23.89 (0.05) | 23.58 (0.13) | 23.07 (0.07) | 28.45 (0.18) | 25.95 (0.00) | 22.96 (0.08) | 24.16 (0.09) | 23.37 (0.17) |
|  | YWHAZ | 18.89 (0.12) | 18.71 (0.06) | 20.69 (0.03) | 18.78 (0.17) | 18.75 (0.02) | 19.30 (0.10) | 20.23 (0.21) | 19.64 (0.19) | 19.50 (1.15) | 22.66 (0.04) | 21.15 (0.13) | 17.93 (0.12) | 19.71 (0.15) | 19.00 (0.04) |
| 5 months | B2M | 19.78 (0.01) | 19.39 (0.06) | 20.02 (0.04) | 19.59 (0.12) | 18.86 (0.03) | 19.67 (0.03) | 19.61 (0.23) | 17.89 (0.05) | 18.56 (0.03) | 35.47 (0.60) | 22.35 (0.24) | 17.83 (0.13) | 21.49 (0.15) | 18.27 (1.13) |
|  | BLM | 23.90 (0.09) | 23.62 (0.05) | 24.59 (0.04) | 24.31 (0.10) | 23.25 (0.07) | 25.00 (0.08) | 25.85 (0.11) | 24.33 (0.09) | 23.58 (0.29) | 33.48 (0.76) | 25.55 (0.11) | 23.90 (0.02) | 24.90 (0.07) | 22.28 (0.17) |
|  | GAPDH | 26.50 (0.12) | 26.16 (0.04) | 21.67 (0.05) | 25.49 (0.04) | 25.51 (0.05) | 23.86 (0.15) | 25.59 (0.15) | 26.08 (0.09) | 24.97 (0.23) | 37.23 (2.62) | 27.04 (0.29) | 25.10 (0.26) | 28.45 (0.09) | 24.36 (0.07) |
|  | HPRT1 | 21.50 (0.30) | 21.73 (0.29) | 22.31 (0.44) | 17.70 (1.66) | 22.05 (0.10) | 20.78 (0.24) | 13.62 (3.06) | 20.67 (0.45) | 21.62 (0.06) | 37.05 (0.01) | 23.48 (0.60) | 18.57 (0.30) | 22.78 (0.16) | 21.24 (0.24) |
|  | PPIA | 16.15 (0.68) | 16.66 (0.13) | 18.14 (0.18) | 17.37 (0.17) | 16.73 (0.28) | 16.08 (0.44) | 16.38 (0.17) | 15.95 (0.42) | 16.26 (0.07) | 31.33 (0.34) | 17.22 (0.13) | 15.50 (0.25) | 16.93 (0.57) | 15.84 (0.99) |
|  | RPL4 | 16.11 (0.23) | 16.11 (0.33) | 16.80 (0.14) | 16.83 (0.14) | 16.36 (0.26) | 16.94 (0.76) | 16.91 (0.44) | 15.85 (0.12) | 15.98 (0.14) | 31.25 (0.68) | 16.97 (0.12) | 15.37 (0.70) | 16.29 (0.64) | 14.64 (0.08) |
|  | SDHA | 21.85 (0.32) | 21.28 (0.09) | 18.56 (0.56) | 21.39 (0.52) | 21.21 (0.34) | 18.48 (0.04) | 20.09 (0.14) | 22.37 (0.25) | 22.28 (0.13) | 35.18 (0.62) | 24.72 (0.28) | 21.62 (0.55) | 22.16 (0.46) | 21.52 (0.17) |
|  | TBP | 23.44 (0.09) | 23.61 (0.14) | 24.14 (0.06) | 24.19 (0.11) | 23.66 (0.16) | 22.90 (0.06) | 24.28 (0.05) | 22.94 (0.04) | 23.48 (0.08) | 33.13 (0.54) | 24.67 (0.09) | 23.34 (0.10) | 23.99 (0.06) | 22.63 (0.12) |
|  | YWHAZ | 18.51 (0.11) | 18.65 (0.16) | 19.97 (0.20) | 19.00 (0.12) | 18.58 (0.28) | 19.05 (0.46) | 21.21 (0.08) | 18.24 (0.45) | 18.59 (0.21) | 31.97 (0.28) | 19.80 (0.10) | 18.58 (0.14) | 19.32 (0.29) | 17.40 (0.16) |
