# Supplementary material for: Age-related changes in relative expression stability of commonly used housekeeping genes in selected porcine tissues
Source: BMC Res Notes. 2011 Oct 24;4:441. doi: 10.1186/1756-0500-4-441 (PMC3219825; doi:10.1186/1756-0500-4-441)
Supplement: Additional file 2 — Relative expression of candidate genes and effect of age and organ on expression level. Overall expression data of reference candidate genes. Summary of the Proc GLM (ver.9.2; SAS, SAS Institute Inc., Cary, NC, USA) analysis detecting significant effects of age, organs and age-organ interaction on the expression of reference candidate genes. ***P<0.001. [file 1756-0500-4-441-S2.DOC]

**Table S2. Relative expression of candidate genes and effect of age and organ on expression level**

**(calculated by PROC GLM)**

| Gene | Mean±SD | Tissue | Age | Tissue*Age | R^2^ | Model |
| --- | --- | --- | --- | --- | --- | --- |
| B2M | 20.03±3.32 | *** | *** | *** | 0.996 | *** |
| BLM | 24.77±2.44 | *** | *** | *** | 0.995 | *** |
| GAPDH | 26.44±2.75 | *** | *** | *** | 0.977 | *** |
| HPRT1 | 22.05±3.82 | *** | *** | *** | 0.981 | *** |
| PPIA | 16.91±3.21 | *** | *** | *** | 0.991 | *** |
| RPL4 | 16.92±3.12 | *** | *** | *** | 0.989 | *** |
| SDHA | 21.17±3.1 | *** | *** | *** | 0.993 | *** |
| TBP | 24.22±2.25 | *** | *** | *** | 0.994 | *** |
| YWHAZ | 19.97±2.96 | *** | *** | *** | 0.995 | *** |

****P* <0.001
